# Factors Influencing Intergovernmental Cooperation on Emission Reduction in Chengdu-Chongqing Urban Agglomeration: An Evolutionary Game Theory Perspective

**DOI:** 10.3390/ijerph192214848

**Published:** 2022-11-11

**Authors:** Jingyu Liu, Weidong Meng, Bo Huang, Yuyu Li

**Affiliations:** 1 School of Economics and Management, Chongqing Normal University, Chongqing 401331, China; 2 School of Economics and Business Administration, Chongqing University, Chongqing 400044, China

**Keywords:** evolutionary game, dynamic carbon tax, regional cooperation, carbon emission, inter-governmental cooperation, Chengdu-Chongqing urban agglomeration

## Abstract

In this study, we introduced the realistic problem of a dynamic carbon tax, built several evolutionary game models for cooperative emission reduction by local governments, and determined the factors that influence governments’ willingness to cooperate in emission reduction. The findings revealed that, first, the probability of governments opting for cooperative emission reduction strategies increases at different rates depending on the benefits and costs of cooperation. Second, externalities influence governments’ willingness to cooperate in emission reduction during cooperative carbon emission reduction. Furthermore, the emergence of a free-riding situation reduces the effectiveness of intergovernmental cooperation in reducing carbon emissions. Third, carbon tax policy can influence the likelihood that local governments will choose cooperative emission reduction, and different carbon tax sizes have different effects on their willingness to choose cooperative emission reduction.

## 1. Introduction

Since the industrial revolution, the rapid growth of the industrial economy has been the primary driving force behind the world economy’s sustained growth. However, it has also resulted in an increase in total carbon dioxide emissions, which has resulted in environmental issues such as global warming, sea level rise, and so on [1]. In this process, China’s economy has developed a broad growth pattern characterized by high input, high consumption, and low output. Coal and oil account for a relatively large proportion of its energy consumption structure, and the intensity of carbon dioxide emissions is high [2]. According to International Energy Agency data, China’s total carbon emissions surpassed those of the United States in 2007, making China the largest contributor to global carbon emissions. Its carbon dioxide emissions accounted for 19.2 percent of total global emissions in 2016 [3]. The Chinese government, as a responsible large country, is aware of the serious environmental problems caused by carbon dioxide emissions. Therefore, at the 75th United Nations General Assembly, President Xi Jinping committed to the international community that China will strive to reach peak carbon emissions by 2030 and achieve carbon neutrality by 2060 [4]. However, China is still in the middle stage of industrialization, and the characteristics of this stage indicate that carbon dioxide emissions will continue to rise rapidly as the economy develops. How to choose a scientific and feasible emission reduction path under the dual constraints of emission reduction targets and pressure is an essential basis for curbing carbon dioxide emissions and reaching the peak ahead of time.

Carbon tax policy, carbon emissions trading, clean development mechanisms, and regional alliance cooperation are common international emission reduction incentive strategies, but not all of them are consistent with China’s social development trend. It is worth noting that the report of the Communist Party of China’s 19th National Congress proposed a strategy of coordinated regional development, implying that regional cooperation and development is an unavoidable trend in China’s economic development. The trend toward cross-administrative regional integration is becoming more visible [5]. At the same time, the indivisibility of the regional environment (negative externality) and the sharing of governance benefits (positive externality) necessitate breaking the original “fragmented” governance pattern of local governments based on traditional administrative jurisdictions. Given the characteristics of China’s social and economic practices, reducing carbon dioxide emissions through regional alliance cooperation in emission reduction is clearly preferable and is also more consistent with the future development trend [6].

The sixth meeting of the Communist Party of China’s Central Committee of Finance and Economics emphasized the importance of strengthening ecological environment protection in order to promote the construction of the Chengdu Chongqing double city economic circle and a strategic highland for inland opening-up. In other words, the Chengdu Chongqing double city economic circle is tackling the difficult task of “exploring a new path for green transformation and development” and “jointly building an ecological barrier in the upper reaches of the Yangtze River” [7,8]. Simultaneously, the complementarity of resources in Chengdu and Chongqing provides a natural impetus and foundation for the two sides’ cooperation in reducing emissions. Chongqing, for example, is rich in forest resources, whereas Sichuan’s forest coverage is lower, and its carbon emissions are twice those of Chongqing; Sichuan is rich in hydropower, whereas Chongqing lacks power resources. Furthermore, Chengdu and Chongqing signed the Strategic Cooperation Agreement of the Chongqing Ecological Environment Bureau and the Chengdu Ecological Environment Bureau, which will lead the country in collaborative carbon emission reduction. Clearly, the Chengdu-Chongqing region has laid the groundwork for regional alliance cooperation and emission reduction from political, economic, and other perspectives. Exploring the Chengdu Chongqing urban agglomeration’s carbon emission reduction path can also serve as a model for other regional alliances.

To summarize, establishing a regional alliance to collaborate in emission reduction is an unavoidable strategic measure to achieve carbon neutrality in China, and the positioning of the Chengdu Chongqing double city economic circle construction and the complementarity of resources between the two sides provide both sides with sufficient power and a solid foundation for emission reduction cooperation. Previous studies, however, paid little attention to each regional subject’s contribution to the achievement of emission reduction goals, and the analysis of the internal relationship between regional economic development and the selection of emission reduction strategies was not systematic enough, lacking targeted and operable research and theoretical guidance. Therefore, based on previous research and using evolutionary game theory, this paper will introduce the factor of dynamic carbon tax policy and simulate the evolution process of strategies with or without carbon tax between two governments and with or without dynamic carbon tax among multiple governments in the region. It will identify the factors influencing governments’ willingness to cooperate in emission reduction and then make policy recommendations to improve the relationship between regional cooperation in emission reduction.

The remainder of this paper is structured as follows: Section 2 introduces relevant literature. Section 3 discusses issues and variables. Section 4 examines both governments’ evolution strategy processes in the absence of a dynamic carbon tax. Section 5 describes the evolution of both governments’ strategies in the case of the dynamic carbon tax. Section 6 describes the process of tripartite government strategy evolution in the absence of a dynamic carbon tax. Section 7 provides a numerical analysis and further discusses the relevant factors influencing government strategy. Section 8 summarizes the evolution process as well as the key elements of intergovernmental cooperation in emission reduction.

## 2. Literature Review

Scholars focus on “regional cooperation” and “regional governance” based on existing research. Foreign scholars began using regional governance theory to study regional economic issues as early as the twentieth century. Later, as more scholars in other fields began to focus on regional governance, four theoretical paradigms of urban regional governance emerged: Metropolitan Government theory [9], Public Choice theory [10], New Regionalism [11], and Re-Regionalization [12]. Based on the practical characteristics of China’s regional development, domestic scholars investigated the main profit-seeking characteristics in the process of regional governance [13,14], interactive relations, and so on, and further introduced transaction cost theory, game theory, and other methods to conduct in-depth research on the necessity of regional cooperation [15,16].

In recent years, the academic community has focused more on prominent environmental issues such as air pollution [17] and how to reduce carbon emissions through regional cooperation. International cooperation to reduce carbon emissions can be accomplished in four ways. One option is to tax carbon emissions in order to internalize their negative externalities. Existing research has found that carbon tax policy can effectively reduce carbon emissions [18,19], and that appropriate carbon tax rates can offset the negative economic impact of taxation [20,21]. Domestic researchers discovered that a carbon tax can aid in the achievement of long-term emission reduction goals and that an appropriate carbon tax can reduce economic losses [22,23,24]. Scholars in China, however, shifted from the national to the regional level due to significant regional differences. They discovered that the carbon tax effect is related to local economic development [25] and that it will cause greater economic losses in underdeveloped regions while benefiting developed regions [26]. Second, carbon emissions trading supplements the surplus and deficit, and regional cooperation and emission reduction are realized through a market-oriented approach. Domestic and international scholars discussed the importance of establishing a carbon emission trading market [27,28,29] as well as potential influencing factors [30]. The third option is to use the clean development mechanism, which is a cooperative mode in which one country invests in emission reduction projects in order to obtain carbon emissions from other countries in order to achieve emission reduction. Academics elaborated on the impact factors of implementing the clean development mechanism [31,32] as well as the impact of industry differences [33]. The fourth step is to form regional alliances in order to achieve total emission reductions through joint implementation. According to existing research, alliances between countries [34] and domestic cities [35] can mitigate the negative effects of carbon emissions.

Domestic and foreign scholars have investigated regional cooperative governance from both theoretical and practical perspectives, concluding that regional cooperative governance is consistent with the characteristics of China’s future development. At the same time, this paper discusses various cooperative emission reduction modes and mechanistic characteristics in order to address the prominent environmental problem of carbon emission reduction. However, regional cooperation on emission reduction is still in its early stages, and research on the subject is limited. Although some scholars have demonstrated that carbon tax policies can effectively reduce carbon emissions, they did not go on to discuss whether carbon tax policies can improve regional government cooperation in carbon emission reduction. Will the central government’s adoption of a dynamic carbon tax policy increase the government’s willingness to choose cooperative emission reduction? Second, this study considers three examples of intergovernmental regional cooperation on emission reduction, which compensate to some extent for the lack of regional cooperation governance research.

## 3. Problem and Variables

The Chengdu-Chongqing Shuangcheng Economic Circle has undertaken the economic mission of building a new pattern of national development. It is also an important undertaking of emission reduction, gradually freeing economic development from the dependence on fossil energy. However, reducing carbon emissions benefits public health and has positive external effects, and the marginal private benefit of reducing carbon emissions is smaller than the marginal social benefit. Without central government constraints, local governments will face insufficient supply and a beggar-thy-neighbor welfare trend in environmental decision-making [36]. The unilateral territorial management model is incapable of producing satisfactory results [37,38]. Although regional cooperation can effectively decrease the spread of air pollution and control transboundary pollution, the local governments cannot cooperate through negotiation due to cooperation costs. Therefore, cooperation can only be adjusted by environmental regulations [39]. Similarly, local governments in the Chengdu-Chongqing economic circle can maximize their benefits by implementing various emission reduction strategies. Local governments, for example, will not choose the cooperative emission reduction strategy on their own because of lower private marginal benefits and higher transaction costs. When only one government chooses the strategy of cooperation, affected by external effects, the party that chooses cooperative emission reduction brings positive external effects to the other government.

We assumed that local government strategies A are (Cooperation, Not cooperation), where the probability of choosing ‘Cooperation’ is x and the probability of choosing ‘Not cooperation’ is 1−x. Similarly, the strategies of local government B are also (Cooperation, Not cooperation), the probability of choosing ‘Cooperation’ is y, and the probability of choosing ‘Not cooperation’ is 1−y.

When local government A and local government B cooperate in emission reduction, local government A chooses the strategy of ’Cooperation’ to bring its revenue $R_{A}$, and incurs a cost $C_{A}$. Additionally, if local government A chooses the strategy of ’Cooperation’, the synergistic revenue is $\pi_{s}$ and the cooperative cost incurred is $C_{s}$.

Similarly, the benefits and costs of cooperative emission reduction by local government B are $R_{B}$ and $C_{B}$, respectively, and the co-benefits and costs are $\pi_{s}$ and $C_{s}$, respectively.

When either local government A or local government B chooses to cooperate in emission reduction, and the other chooses the strategy of ‘Not cooperation’, the party that chooses to cooperate in emission reduction brings positive external effects to the other.

For example, when local government A chooses the strategy of ‘Cooperation’, it brings positive benefits of $\theta_{A}$ to local government B. On the contrary, when local government B chooses the strategy of ‘Cooperation’, it brings positive benefits of $\theta_{B}$ to local government A.

Furthermore, if the central government implements the dynamic carbon tax policy and the local government does not implement the cooperative emission reduction strategy, a carbon tax will be imposed. Table 1 shows the specifics:

## 4. Situation without Dynamic Carbon tax

### 4.1. Basic Model

According to Table 2, we expressed the expected payoffs of the strategies of ‘Cooperation’ $\pi_{0}^{x}$ and ‘Not Cooperation’ $\pi_{0}^{1-x}$, and their average profits ${\bar{\pi }}_{0}$ adopted by the local governments as follows

$$\pi_{0}^{x}=y\left(R_{A}-C_{A}+\pi_{s}-C_{s}\right)+\left(1-y\right)\left(R_{A}-C_{A}-\theta_{B}\right)$$

$$\pi_{0}^{1-x}=y\left(\theta_{B}\right)+\left(1-y\right)\left(-\theta_{B}\right)$$

$${\bar{\pi }}_{0*}=x\pi_{0}^{x}+\left(1-x\right)\pi_{0}^{1-x}$$

Therefore, the replication dynamic equation of the choice of the local government is represented as follows:

(1) $$F_{0}(x)=\frac{dx}{dt}=x\left(1-x\right)\left(R_{A}-C_{A}^{}-C_{s}y-\theta_{B}y+\pi_{s}y\right)$$

Similarly, the expected profits of the local governments from ‘Cooperation’ and ‘Not Cooperation’ can be expressed as $\pi_{0}^{y}$ and $\pi_{0}^{1-y}$, respectively, and the average profits can be expressed as ${\bar{\pi }}_{0}$.

$$\pi_{0}^{y}=x\left(R_{B}-C_{B}+\pi_{s}-C_{s}\right)+\left(1-x\right)\left(R_{B}-C_{B}-\theta_{B}\right)$$

$$\pi_{0}^{1-y}=x\left(\theta_{A}\right)+\left(1-x\right)\left(-\theta_{A}\right)$$

$${\bar{\pi }}_{0**}=y\pi_{0}^{y}+\left(1-y\right)\pi_{0}^{1-y}$$

The replication dynamic equation of local government B is represented as follows:

(2) $$F_{0}(y)=\frac{dy}{dt}=y\left(1-y\right)(\pi_{}^{y}-\pi_{}^{1-y})=y\left(1-y\right)\left(R_{B}-C_{B}^{}-C_{s}x-\theta_{A}x+\pi_{s}x\right)$$

### 4.2. Model Analysis

We formulated a simultaneous equation using Equations (1) and (2). We determined that the five equilibrium points of the evolutionary game were: (1,1), (0,0), (0,1), (1,0), and $\left(x_{0},y_{0}\right)$, where $0\leq x_{0}\leq 1$ and $0\leq y_{0}\leq 1$. Next, we obtained the fifth equilibrium point (x,y): $x_{0}=\frac{R_{B}-C_{B}^{}}{C_{s}+\theta_{A}-\pi_{s}}$, $y_{0}=\frac{R_{A}-C_{A}^{}}{C_{s}+\theta_{B}-\pi_{s}}$.

Based on the local stability analysis method for testing the properties of equilibrium points proposed by Fredman [40], the stability of the equilibrium points was obtained from the local stability analysis of the system’s Jacobian matrix.

If the determinant of the equilibrium Jacobian matrix $Det(J_{*})>0$ and $Tr(J_{*})<0$, the corresponding equilibrium point has the property of gradual stability, also known as the *ESS* (Evolutionary Stable Strategy). Therefore, the Jacobian matrix of the evolution system is as follows:

$$\begin{array}{l} J_{0}=\left[\begin{array}{cc} \frac{\partial F_{0}\left(x\right)}{\partial x} & \frac{\partial F_{0}\left(x\right)}{\partial y}\\ \frac{\partial F_{0}\left(y\right)}{\partial x} & \frac{\partial F_{0}\left(y\right)}{\partial y} \end{array}\right]\\ \begin{array}{cc} & =\left[\begin{array}{cc} \left(1-2x\right)\left(R_{A}-C_{A}^{}-C_{s}y-\theta_{B}y+\pi_{s}y\right) & x\left(1-x\right)\left(\pi_{s}-\theta_{B}-C_{s}\right)\\ y\left(1-y\right)\left(\pi_{s}-\theta_{A}-C_{s}\right) & \left(1-2y\right)\left(R_{B}-C_{B}^{}-C_{s}x-\theta_{A}x+\pi_{s}x\right) \end{array}\right] \end{array} \end{array}$$

Finally, the local stability of each equilibrium point under the initial state of different parameters is obtained using the discrimination method. Thus, there are four systematic cases regarding the choice of strategy for local government A and local government B, which are represented as follows:

System I: When both conditions $R_{A}-C_{A}^{}+\pi_{s}-C_{s}-\theta_{B}<0$ and $R_{B}-C_{B}^{}+\pi_{s}-C_{s}x-\theta_{A}<0$ are satisfied, two equilibrium points are present simultaneously. These equilibrium points are (1,0) and (1,1).

System II: When both conditions $R_{A}-C_{A}^{}+\pi_{s}-C_{s}-\theta_{B}>0$ and $R_{B}-C_{B}^{}+\pi_{s}-C_{s}x-\theta_{A}<0$ are satisfied, only one equilibrium point is present (1,0).

System III: When both conditions $R_{A}-C_{A}^{}+\pi_{s}-C_{s}-\theta_{B}<0$ and $R_{B}-C_{B}^{}+\pi_{s}-C_{s}x-\theta_{A}>0$ are satisfied, only one equilibrium point is present (0,1).

System IV: When both conditions $R_{A}-C_{A}^{}+\pi_{s}-C_{s}-\theta_{B}>0$ and $R_{B}-C_{B}^{}+\pi_{s}-C_{s}x-\theta_{A}>0$ are satisfied, only one equilibrium point is present (1,1).

## 5. Situation with Dynamic Carbon Tax

### 5.1. Basic Model

A carbon tax policy is critical for China’s goal of carbon peaking. It has the potential to significantly improve the efficiency of carbon emission reduction. However, it is debatable whether the carbon tax policy can motivate intergovernmental cooperation. Studies should be conducted to determine whether the dynamic carbon tax policy can also increase the willingness of governments to cooperate. Therefore, without the loss of generality, we consider that the value of the dynamic carbon tax is positively correlated with the probability that the local government chooses the strategy of ‘Not cooperation’, i.e., F(1−x) and F(1−y).

According to Table 3, $\pi_{1}^{x}$ and $\pi_{1}^{1-x}$ were the expected benefits for the local government A derived from selecting the ‘Cooperation’ and ‘Not cooperation’ strategies, respectively, calculated using the following formulae:

$$\pi_{1}^{x}=y\left(R_{A}-C_{A}+\pi_{s}-C_{s}\right)+\left(1-y\right)\left(R_{A}-C_{A}-\theta_{B}\right)$$

$$\pi_{1}^{1-x}=y\left(\theta_{B}-F(1-x)\right)+\left(1-y\right)\left(-\theta_{B}-F(1-x)\right)$$

The average expected benefit of the mixed strategy adopted by the local government A is given as follows:

$${\bar{\pi }}_{1*}=x\pi_{1}^{x}+\left(1-x\right)\pi_{1}^{1-x}$$

The replication dynamic equation of the local government A is represented as follows:

(3) $$F_{1}(x)=\frac{dx}{dt}=x\left(1-x\right)\left(R_{A}-C_{A}^{}+F-C_{s}y-\theta_{B}y+\pi_{s}y-Fx\right)$$

$\pi_{1}^{y}$ and $\pi_{1}^{1-y}$ represented the expected benefits of the local government B when it adopts the ‘Cooperation’ and ‘Not cooperation’ strategies, respectively, and can be calculated as follows:

$$\pi_{1}^{y}=x\left(R_{B}-C_{B}+\pi_{s}-C_{s}\right)+\left(1-x\right)\left(R_{B}-C_{B}-\theta_{A}\right)$$

$$\pi_{1}^{1-y}=x\left(\theta_{B}-F(1-y)\right)+\left(1-x\right)\left(-\theta_{A}-F(1-y)\right)$$

The average expected return of the mixed strategy can be calculated as follows:

$${\bar{\pi }}_{1**}=y\pi_{1}^{y}+\left(1-y\right)\pi_{1}^{1-y}$$

The replication dynamics equation of the local government B is shown in Equation (4)

(4) $$F_{1}(y)=\frac{dy}{dt}=y\left(1-y\right)(\pi_{t}^{y}-\pi_{t}^{1-y})=y\left(1-y\right)\left(R_{B}-C_{B}^{}+F-C_{s}x-\theta_{A}x+\pi_{s}x-Fy\right)$$

### 5.2. Model Analysis

Similarly, according to Equations (3) and (4), the five systematic partial equilibriums are (1,1), (0,0), (0,1), (1,0), and $\left(x_{1},y_{1}\right)$, where $0\leq x_{1}\leq 1$ and $0\leq y_{1}\leq 1$. If the determinant of the equilibrium Jacobian matrix $Det(J_{1})>0$ and $Tr(J_{1})<0$ [40], the corresponding equilibrium point has the property of gradual stability, also known as the *ESS* (Evolutionary Stable Strategy). The Jacobian matrix of the evolution system can be expressed as follows:

$$\begin{array}{l} J_{1}=\left[\begin{array}{cc} \frac{\partial F_{1}\left(x\right)}{\partial x} & \frac{\partial F_{1}\left(x\right)}{\partial y}\\ \frac{\partial F_{1}\left(y\right)}{\partial x} & \frac{\partial F_{1}\left(y\right)}{\partial y} \end{array}\right]\\ \begin{array}{cc} & =[\begin{array}{c} \left(1-2x\right)\left(R_{A}-C_{A}^{}+F-C_{s}y-\theta_{B}y+\pi_{s}y-Fx\right)-Fx\left(1-x\right)\\ y\left(1-y\right)\left(\pi_{s}-C_{s}-\theta_{A}\right) \end{array} \end{array}\begin{array}{c} x\left(1-x\right)\left(\pi_{s}-C_{s}-\theta_{B}\right)\\ \left(1-2y\right)\left(R_{B}-C_{B}^{}+F-C_{s}x-\theta_{A}x+\pi_{s}x-Fy\right)-Fy\left(1-y\right) \end{array}] \end{array}$$

The expressions of the Jacobian matrix $Det(J_{1})>0$ and $Tr(J_{1})<0$ for each equilibrium point are shown in Table 4. Finally, using the discrimination method, the local stability of each equilibrium point under the initial state of different parameters is determined. For simplicity, we assume that $g_{1}=R_{A}-C_{A}^{}+\pi_{s}-C_{s}-\theta_{B}$ and $g_{2}=R_{B}-C_{B}^{}+\pi_{s}-C_{s}-\theta_{A}$.

Thus, there are four systematic cases regarding the choice of strategy for the local government A and the local government B, which are represented as follows:

**System I:** The equilibrium points (0,1) and (1,0) are ESS if Condition 1 is satisfied.

**System II:** The equilibrium point (0,1) is an ESS if Conditions 2 and 3 are satisfied.

**System III:** The equilibrium point (1,0) is an ESS if Conditions 4 and 7 are satisfied.

**System IV:** The equilibrium points are not in stable equilibrium if Conditions 5, 6, and 8 are satisfied.

**System V:** The equilibrium point (1,1) is ESS if Condition 9 is satisfied.

We defined another equilibrium point $\left(x_{*},y_{*}\right)$, *=0,1, in two situations, i.e., without a carbon tax and with a carbon tax. In different situations, the solutions specified in Equations (5) and (6) are as follows:

(5) $$\left(x_{0},y_{0}\right)=\left(\frac{R_{B}-C_{B}^{}}{C_{s}+\theta_{A}-\pi_{s}},\frac{R_{A}-C_{A}^{}}{C_{s}+\theta_{B}-\pi_{s}}\right)$$

(6) $$\left(x_{1},y_{1}\right)=\left(\frac{\Delta_{1}F^{2}+g_{3}\Delta_{2}F}{g_{3}\left(F^{2}-g_{3}g_{4}\right)}-\Delta_{1},\frac{\Delta_{1}F+g_{3}\Delta_{2}}{F^{2}-g_{3}g_{4}}\right)$$

Here, $\Delta_{1}=R_{B}-C_{B}+F$, $\Delta_{2}=R_{A}-C_{A}+F$, $g_{3}=\pi_{s}-C_{s}-\theta_{A}$ and $g_{4}=\pi_{s}-C_{s}-\theta_{B}$.

The conditions $0\leq x_{*}\leq 1$ and $0\leq y_{*}\leq 1$, *=0,1, should also be satisfied to ensure $\left(x_{*},y_{*}\right)\in \left[0,1\right]\times \left[0,1\right]$.

### 5.3. Factor Analysis of System I under Different Situations

**Figure 1:**
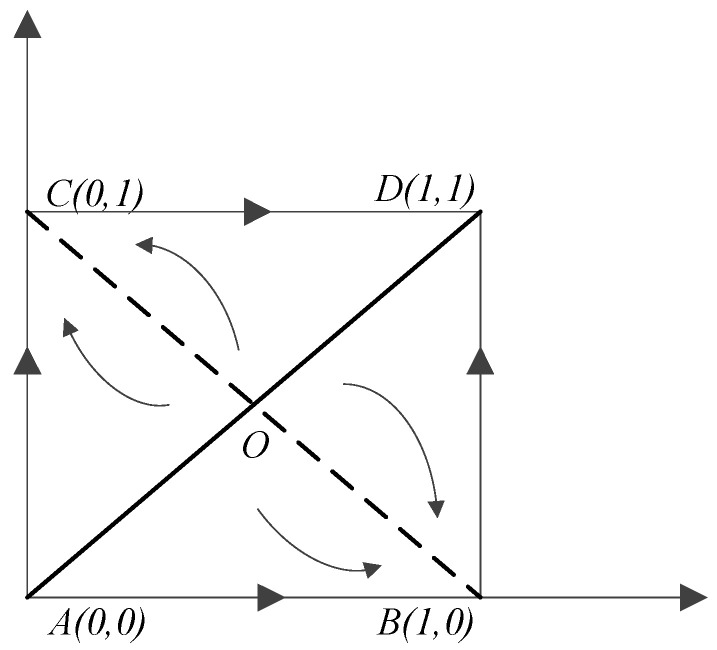
Replicated dynamic phase diagram of system I.

In Figure 1, the evolutionary process and stable state of governmental departments are affected by the initial state of the system (the proportion of different strategies selected in government A and government B) and the relative position of the saddle point O. When the initial state falls in the ACDO region, the evolutionary game system converges to (0,1), and the stable strategy gradually chooses cooperative emission reduction from one side, and the non-cooperative emission reduction from one side becomes the only ESS. When the initial state falls in the ABDO region, the evolutionary game system converges to (1,0), and the stable strategy gradually evolves in the direction of the cooperative emission reduction of one party and the non-cooperative emission reduction of the other party.

The local government game’s long-term equilibrium result revealed that one side cooperates in emission reduction while the other does not. Its specific evolutionary path and stability depend on the size of the regional areas ABDO and ACDO. Therefore, government A and government B might evolve in two directions: when the system reaches stability, one of the two governments will choose the “cooperative emission reduction” strategy.

Taking the analysis $S_{ACDO}^{*}$ as an example, the probability of the system evolving a stable strategy (0,1) with different penalties can be calculated as follows:

$$S_{ACDO}^{0}=\frac{1}{2}\left(x_{0}-y_{0}+1\right)=\frac{1}{2}\left(\frac{R_{B}-C_{B}^{}}{C_{s}+\theta_{A}-\pi_{s}}-\frac{R_{A}-C_{A}^{}}{C_{s}+\theta_{B}-\pi_{s}}+1\right)$$

$$S_{ACDO}^{1}=\frac{1}{2}\left(x_{1}-y_{1}+1\right)=\frac{1}{2}\left(\frac{\Delta_{1}F^{2}+g_{3}\Delta_{2}F}{g_{3}\left(F^{2}-g_{3}g_{4}\right)}-\frac{\Delta_{1}}{g_{3}}-\frac{\Delta_{1}F+g_{3}\Delta_{2}}{F^{2}-g_{3}g_{4}}+1\right)$$

(1) Influence of F on the strategies of the government

Based on the two situations, taking the first derivative of $S_{ACDO}^{1}$ with respect to F, we can obtain $\partial S_{ACDO}^{1}/\partial F<0$. According to the results, the slope is negative, indicating that as the value of the dynamic carbon tax given by the central government increases, the value of $S_{ACDO}^{1}$ decreases, and the probability that the initial probability of cooperative emission reduction strategy chosen by each government will fall in the region ABDO increases, i.e., the probability of both sides tending to (1,0) increases. When the system is stable, one government will opt for a “non-cooperative emission reduction” strategy, while another will opt for a “cooperative emission reduction” strategy. The only option is the evolutionary stability strategy. Thus, whether or not there is a carbon tax policy, one party of the government-to-government cooperation alliance must implement emission reduction measures.

(2) Influence of $\theta_{A}$ and $\theta_{B}$ on the strategies of the government

Taking the first derivative of $S_{ACDO}^{*}$ with respect to $\theta_{A}$ and F, we can obtain $\partial S_{ACDO}^{*}/\partial \theta_{A}^{}<0$ and $\partial S_{ACDO}^{*}/\partial \theta_{B}^{}>0$. The effect of intergovernmental externalities is relative. As $\theta_{A}$ increases and the area of $S_{ACDO}^{*}$ decreases, the probability of the initial probability of local government A falling in the region ACDO decreases, i.e., the probability of (1,0) increases. On the contrary, for F, the probability that the initial probability of local government B falls in the region ACDO increases with the increase in F, and the probability that the strategy tends to (0,1) increases. Externalities have an impact on intergovernmental cooperation to reduce emissions. As one government’s positive externality increases, another’s willingness to choose cooperative emission reduction decreases. Thus, during the cooperation between governments in reducing emissions, the emergence of externalities reduces the efficiency of bilateral cooperation in emission reduction. Therefore, to a certain extent, the central government can provide government subsidies with higher positive externalities to improve its willingness to cooperate in emission reduction. Simultaneously, the “free rider” government is given some sort of punishment mechanism to discourage its desire to choose “non-cooperation emission reduction”.

(3) Influence of $\pi_{s}$ on the strategies of the government

Taking the first derivative of $S_{ACDO}^{*}$ with respect to $\pi_{s}$, we can obtain $\partial S_{ACDO}^{0}/\partial \pi_{s}<0$ and $\partial S_{ACDO}^{1}/\partial \pi_{s}<0$. The formula shows that as the cooperative income of local governments increases, the area of $S_{ACDO}^{*}$ decreases, and the probability that the initial probability of each local government falls in the region ACDO also decreases, i.e., the probability that the strategy tends to (1,0) increases.

## 6. Situation with Three Cities and No Dynamic Carbon Tax

### 6.1. Basic Model

In 2021, Chengdu Chongqing officially signed the Cooperation Agreement on Establishing Regional Environmental Access Negotiation Mechanism, which jointly aims to build a regional carbon emission trading market to reduce pollution and carbon. Chengdu and Chongqing are the economic centers of the Chengdu Chongqing dual city economic circle, with “dual-core” characteristics, as China’s fourth pole of growth. Therefore, hot spots in terms of total carbon emissions and average land carbon emissions are concentrated in the two cities [41]. As Chengdu and Chongqing jointly promote co-construction and co-insurance, it is important to determine ways for these cities to perform cooperative emission reduction, considering that both have high carbon emissions.

**Figure 2:**
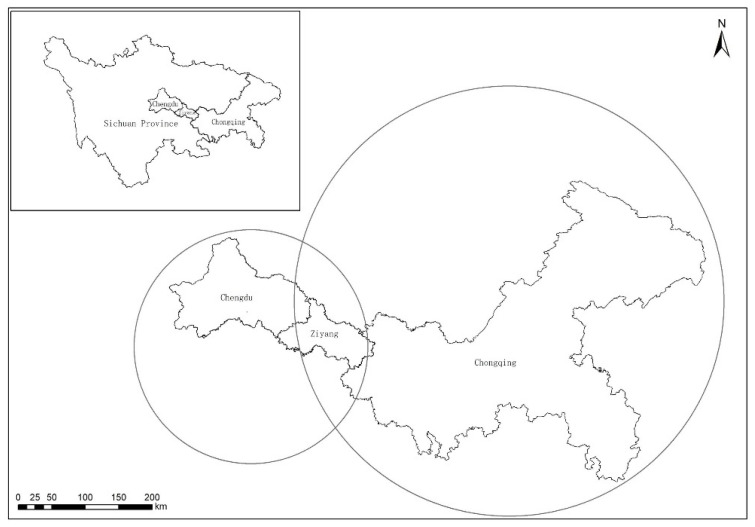
Urban location map.

However, since Chengdu and Chongqing are not adjacent, Ziyang is introduced in this part to simulate cooperative emission reduction among the three cities (Figure 2). Additionally, since the impact of a dynamic carbon tax on the three cities is similar to that on the two cities, this part only considers the situation without the dynamic carbon tax. Due to the existence of marginal diminishing effects in tripartite cooperative emission reduction, when the tripartite cooperative emission reduction coincides, the cooperative emission reduction benefits of the intermediate city (Ziyang City) are not the sum of the positive external effects of the neighboring cities. Thus, it is assumed that γ is the marginal decreasing effect factor, where γ∈(0,1).

According to Table 5, we expressed the expected payoffs of the strategies of the local governments ‘Cooperation’ $\pi_{2}^{x}$, ‘Not Cooperation’ $\pi_{2}^{1-x}$, and their average profits ${\bar{\pi }}_{2*}$ as follows:

$$\begin{array}{l} \pi_{2}^{x}=yz\left(R_{A}-C_{A}+\pi_{s}-C_{s}\right)+y\left(1-z\right)\left(R_{A}-C_{A}+\pi_{s}-C_{s}\right)\\ \;+z\left(1-y\right)\left(R_{A}-C_{A}-\theta_{B}\right)+\left(1-z\right)\left(1-y\right)\left(R_{A}-C_{A}-\theta_{B}\right) \end{array}$$

$$\pi_{2}^{1-x}=yz\left(\theta_{B}\right)+y\left(1-z\right)\left(\theta_{B}\right)+z\left(1-y\right)\left(-\theta_{B}\right)+\left(1-z\right)\left(1-y\right)\left(-\theta_{B}\right)$$

$${\bar{\pi }}_{2*}=x\pi_{2}^{x}+\left(1-x\right)\pi_{2}^{1-x}$$

Therefore, the replication dynamic equation for local government selection is as follows:

(7) $$F_{2}(x)=\frac{dx}{dt}=x\left(1-x\right)\left(R_{A}-C_{A}^{}-C_{s}y-\theta_{B}y+\pi_{s}y\right)$$

Similarly, the expected profits of the local governments through ‘Cooperation’ and ‘Not Cooperation’, and the average profit are expressed as $\pi_{2}^{y}$, $\pi_{2}^{1-y}$, and ${\bar{\pi }}_{2**}$, respectively.

$$\begin{array}{l} \pi_{2}^{y}=xz\left(R_{B}-C_{B}+\gamma \left(\pi_{s}-C_{s}\right)\right)+x\left(1-z\right)\left(R_{B}-C_{B}+\pi_{s}-C_{s}-\theta_{C}\right)\\ +z\left(1-x\right)\left(R_{B}-C_{B}+\pi_{s}-C_{s}-\theta_{A}\right)+\left(1-x\right)\left(1-z\right)\left(R_{B}-C_{B}-\theta_{C}-\theta_{A}\right) \end{array}$$

$$\pi_{2}^{1-y}=xz\left(\theta_{C}+\theta_{A}\right)+x\left(1-z\right)\left(\theta_{A}-\theta_{C}\right)+\left(1-x\right)z\left(\theta_{C}-\theta_{A}\right)+\left(1-x\right)\left(1-z\right)\left(-\theta_{C}-\theta_{A}\right)$$

$${\bar{\pi }}_{2**}=y\pi_{2}^{y}+\left(1-y\right)\pi_{2}^{1-y}$$

Hence, the replication dynamic equation of local government B is represented as follows:

(8) $$F_{2}(y)=\frac{dy}{dt}=y\left(1-y\right)(\pi_{2}^{y}-\pi_{2}^{1-y})=y\left(1-y\right)\left(\begin{array}{l} R_{B}-C_{B}^{}-C_{s}x-C_{s}z+\pi_{s}x+\pi_{s}z-\theta_{A}x\\ -\theta_{C}z+2C_{s}xz-2\pi_{s}xz-\gamma C_{s}xz+\gamma \pi_{s}xz \end{array}\right)$$

Similarly, the expected profits of the local governments through ‘Cooperation’ and ‘Not Cooperation’, and the average profit are expressed as $\pi_{2}^{z}$, $\pi_{2}^{1-z}$, and ${\bar{\pi }}_{2***}$, respectively.

$$\begin{array}{l} \pi_{2}^{z}=xy\left(R_{C}-C_{C}+\pi_{s}-C_{s}\right)+x\left(1-y\right)\left(R_{C}-C_{C}-\theta_{B}\right)\\ +y\left(1-x\right)\left(R_{C}-C_{C}+\pi_{s}-C_{s}\right)+\left(1-x\right)\left(1-y\right)\left(R_{C}-C_{C}-\theta_{B}\right) \end{array}$$

$$\pi_{2}^{1-z}=xy\left(\theta_{B}\right)+x\left(1-y\right)\left(-\theta_{B}\right)+y\left(1-x\right)\left(\theta_{B}\right)+\left(1-y\right)\left(1-x\right)\left(-\theta_{B}\right)$$

$${\bar{\pi }}_{2***}=z\pi_{2}^{z}+\left(1-z\right)\pi_{2}^{1-z}$$

Hence, the replication dynamic equation of local government C is represented as follows:

(9) $$F_{2}\left(z\right)=\frac{dz}{dt}=z\left(1-z\right)(\pi_{}^{z}-\pi_{}^{1-z})=z\left(1-z\right)\left(R_{C}-C_{C}^{}-C_{s}y-\theta_{B}y+\pi_{s}y\right)$$

### 6.2. Model Analysis

Let $F_{2}\left(x\right)=F_{2}\left(y\right)=F_{2}\left(z\right)=0$. We can get nine equilibrium points of the copied dynamic equation (0,0,0), (0,0,1), (0,1,0), (1,0,0), (0,1,1), (1,1,0), (1,0,1), (1,1,1), and $\left(x_{2},y_{2},z_{2}\right)$. Among them, $\left(x_{2},y_{2},z_{2}\right)$ represents possible policy solutions, including pure policy solutions and mixed policy solutions. Since the combination of strategies in an asymmetric evolutionary game is an evolutionary stable equilibrium point, this equilibrium is a strict Nash equilibrium. Therefore, $\left(x_{2},y_{2},z_{2}\right)$ will not be discussed.

To analyze the stability of equilibrium points, the Jacobian matrix is constructed first, and then the eigenvalues of each equilibrium point are solved. If all three eigenvalues are negative, it is an ESS. If all three eigenvalues are positive, it is an unstable point. If there are one or two positive values, it is a saddle point. The analysis is as follows:

$$\begin{array}{l} J_{2}=\left[\begin{array}{ccc} \frac{\partial F_{2}\left(x\right)}{\partial x} & \frac{\partial F_{2}\left(x\right)}{\partial y} & \frac{\partial F_{2}\left(x\right)}{\partial z}\\ \frac{\partial F_{2}\left(y\right)}{\partial x} & \frac{\partial F_{2}\left(y\right)}{\partial y} & \frac{\partial F_{2}\left(y\right)}{\partial z}\\ \frac{\partial F_{2}\left(z\right)}{\partial x} & \frac{\partial F_{2}\left(z\right)}{\partial y} & \frac{\partial F_{2}\left(z\right)}{\partial z} \end{array}\right]\\ \begin{array}{cc} & \begin{array}{l} =[\begin{array}{cc} \left(1-2x\right)\left(R_{A}-C_{A}^{}-C_{s}y-\theta_{B}y+\pi_{s}y\right) & x\left(1-x\right)\left(\pi_{s}-\theta_{B}-C_{s}\right)\\ y\left(1-y\right)\left(\pi_{s}-\theta_{A}-C_{s}+2zC_{s}-2z\pi_{s}-\gamma zC_{s}+\gamma z\pi_{s}\right) & \left(1-2y\right)\left(\begin{array}{l} R_{B}-C_{B}^{}-C_{s}x-C_{s}z+\pi_{s}x+\pi_{s}z-\theta_{A}x\\ -\theta_{C}z+2C_{s}xz-2\pi_{s}xz-\gamma C_{s}xz+\gamma \pi_{s}xz \end{array}\right)\\ 0 & z\left(1-z\right)\left(\pi_{s}-\theta_{B}-C_{s}\right) \end{array}\\ \begin{array}{c} 0\\ y\left(1-y\right)\left(\pi_{s}-\theta_{C}-C_{s}+2xC_{s}-2x\pi_{s}-\gamma xC_{s}+\gamma x\pi_{s}\right)\\ \left(1-2z\right)\left(R_{C}-C_{C}^{}-C_{s}y-\theta_{B}y+\pi_{s}y\right) \end{array}] \end{array} \end{array} \end{array}$$

First, the equilibrium point (0,0,0) is analyzed and then it is substituted to obtain the Jacobian matrix:

$$J_{\left(0,0,0\right)}=\left[\begin{array}{ccc} \left(R_{A}-C_{A}^{}\right) & 0 & 0\\ 0 & \left(R_{B}-C_{B}^{}\right) & 0\\ 0 & 0 & \left(R_{C}-C_{C}^{}\right) \end{array}\right]$$

The solution is $\lambda_{1}=R_{A}-C_{A}^{}$, $\lambda_{2}=R_{B}-C_{B}^{}$, and $\lambda_{3}=R_{C}-C_{C}^{}$. According to the previous analysis, (0,0,0) is an unstable point. Similarly, the remaining equilibrium points can be analyzed, and the results of the analysis are shown in Table 6:

As shown in case 5, if $\lambda_{1}=R_{A}-C_{A}^{}+\pi_{s}-C_{s}-\theta_{B}<0$, $\lambda_{2}=-\left[R_{B}-C_{B}^{}-\theta_{A}+\pi_{s}-C_{s}\right]<0$, and $\lambda_{3}=-\left[R_{C}-C_{C}^{}+\pi_{s}-C_{s}-\theta_{B}\right]<0$ exist simultaneously, an equilibrium point (0,1,1) exists. As shown in case 6, if $\lambda_{1}=-\left[R_{A}-C_{A}^{}+\pi_{s}-C_{s}-\theta_{B}\right]<0$,$\lambda_{2}=-\left[R_{B}-C_{B}^{}-\theta_{A}+\pi_{s}-C_{s}\right]<0$, and $\lambda_{3}=R_{C}-C_{C}^{}+\pi_{s}-C_{s}-\theta_{B}<0$ exist simultaneously, an equilibrium point (1,1,0) exists. As shown in case 7, if $\lambda_{2}=R_{B}-C_{B}^{}-\theta_{A}-\theta_{C}+\gamma \left(\pi_{s}-C_{s}\right)<0$ exists, an equilibrium point (1,0,1) exists. As shown in case 8, if $\lambda_{1}=-\left[R_{A}-C_{A}^{}+\pi_{s}-C_{s}-\theta_{B}\right]<0$, $\lambda_{2}=-\left[R_{B}-C_{B}^{}-\theta_{A}-\theta_{C}+\gamma \left(\pi_{s}-C_{s}\right)\right]<0$, and $\lambda_{3}=-\left[R_{C}-C_{C}^{}+\pi_{s}-C_{s}-\theta_{B}\right]<0$ are satisfied simultaneously, a stable point (1,1,1) exists. To summarize, in the cooperative emission reduction alliance of local governments, if the government chooses the cooperative emission reduction strategy to bring more benefits than the negative externalities in the cooperative emission reduction, the government will inevitably choose the cooperative emission reduction strategy.

## 7. Numerical Simulation

To identify the factors affecting the choice of cooperative emission reduction strategy of the local governments more intuitively and determine whether the dynamic carbon tax can further enhance the emission reduction incentive and cooperation willingness of the local governments, MATLAB simulations were performed to analyze which strategy the government might implement and the influencing factors under different parameters.

The Chengdu Chongqing double city economic circle, as the “fourth pole of China’s economy” takes on the major economic mission of the new development pattern as well as the difficult task of reducing emissions. The complementarity of resources between Chengdu and Chongqing provides a solid foundation for cooperation on emission reduction. For example, Chongqing is rich in forest resources, and the forest coverage in Chengdu is lower than that in Chongqing. Without losing generality, it is assumed that the positive externalities brought by the cooperative emission reduction in Chongqing are higher [41], i.e., $\theta_{A}=9$ and $\theta_{B}=8$. Also, considering that the emission reduction technologies and other conditions are the same, without losing generality, we assumed that $R_{A}=6$, $R_{B}=5$, $C_{A}^{}=4$, $C_{B}^{}=3$, $\pi_{s}=7$, $C_{s}=4$, and F=2. Thus, we investigated the influence of different factors on government strategies in System I.

(1) Influence of F on the strategies of local governments

**Figure 3:**
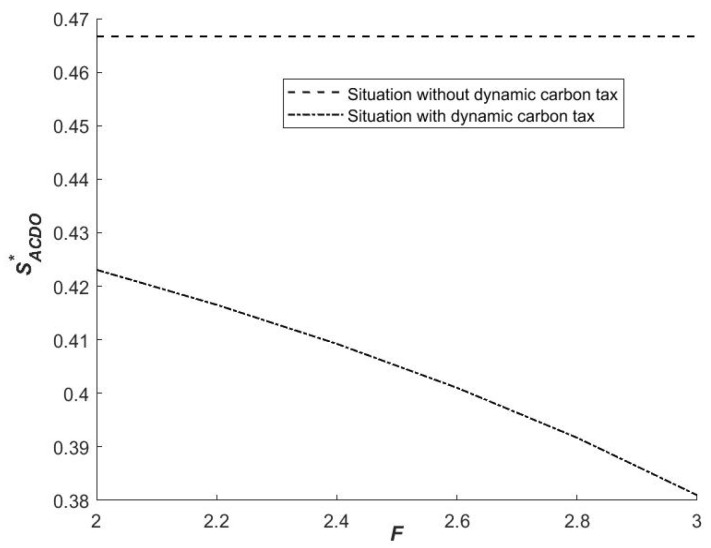
Influence of F on $S_{ACDO}^{*}$.

As shown in Figure 3, as F increases, the size of $S_{ACDO}^{*}$ decreases, and the probability of local governments choosing (1,0) increases, i.e., local government A is more willing to adopt the strategy of ’Cooperation’. At this time, local government A with high resource endowment will still choose cooperative emission reduction. Although it has a high positive external effect on neighboring governments when choosing a cooperative emission reduction strategy, its net benefits are still greater than accrue to those who do not use a cooperative emission reduction strategy. At the same time, the net benefits of local governments that do not choose a cooperative emission reduction strategy will decrease as the severity of the punishment increases. Local government A believes that increasing the severity of punishment will cause neighboring governments to change their strategic choices, resulting in a decrease in interests. For these reasons, the local government A opts for a collaborative emission reduction strategy with increased penalties. To avoid reducing the government’s willingness to choose cooperative emission reduction, the central government can appropriately provide carbon emission reduction subsidies to the party that chooses cooperative emission reduction during this process.

Under the influence of a carbon tax, local government B is still willing to choose the strategy of ‘Not Cooperation,’ implying that the benefit brought by the free-riding of government B is significantly greater than the cost of the dynamic carbon tax (Figure 3). This indicates that the penalty is still low, and the net benefits of non-cooperative emission reduction strategies are still greater than those of cooperative emission reduction strategies. To put it another way, the central government should increase the penalties for governments that do not cooperate to reduce emissions.

(2) Influence of $\theta_{A}$ and $\theta_{B}$ on the strategies of local governments

**Figure 4:**
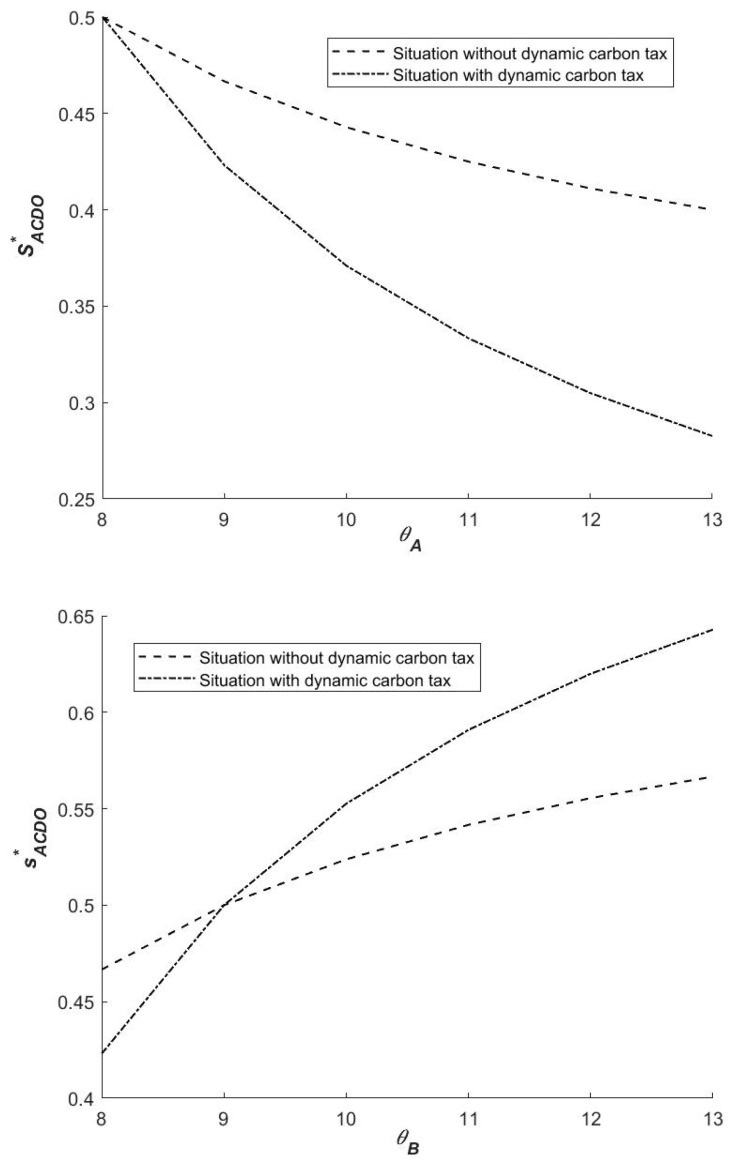
Influence of $\theta_{A}$ and $\theta_{B}$ on $S_{ACDO}^{*}$.

As illustrated in Figure 4, the size of $S_{ACDO}^{*}$ changes in different directions with the increase in $\theta_{A}$ and $\theta_{B}$. This is because $\theta_{A}$ and $\theta_{B}$ originate from different local governments. Because of the different values of $\theta_{A}$ and $\theta_{B}$, the impact of externalities on the selection of local government strategies varies. When $\theta_{A}$ increases, $S_{ACDO}^{*}$ decreases accordingly, and both governments are more likely to choose (1,0). When $\theta_{B}$ increases, $S_{ACDO}^{*}$ increases, and both governments are more inclined to choose (0,1). When the profit from local government A increases, the local government B is more willing to adopt the ‘not cooperation’ strategy. Otherwise, local government A is more likely to adopt the strategy. Regardless of which government chooses a cooperative emission reduction strategy, as the positive external effect increases, the benefits of non-cooperative carbon emission reduction strategies increase, as does the probability of non-cooperation. In other words, when a government’s external effect exceeds its cost (including the cost of strategy selection and external penalty cost), it will inevitably choose not to cooperate in emission reduction. Therefore, the likelihood of choosing non-cooperative emission reduction can be increased in order to improve local governments’ willingness to cooperate in emission reduction. In other words, through carbon subsidies or other legal and political means, the central government should compensate governments that actively participate in reducing carbon emissions.

(3) Influence of $\pi_{S}$ on the strategies of local governments

**Figure 5:**
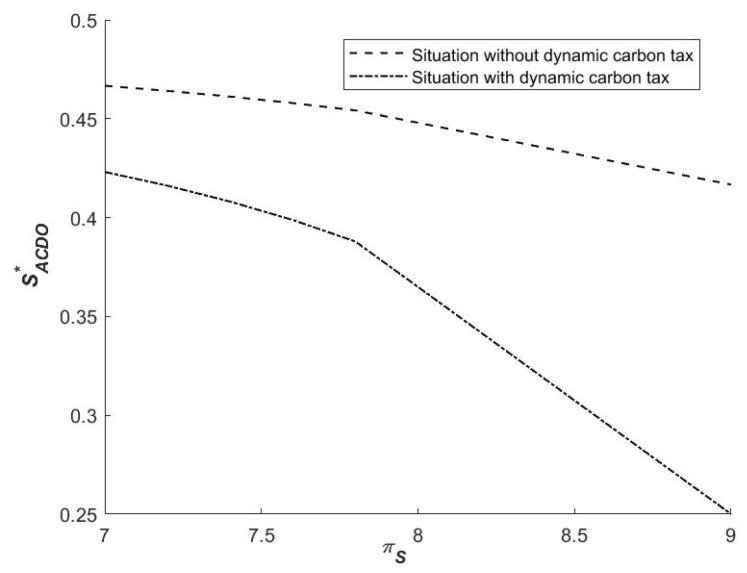
Influence of $\pi_{S}$ on $S_{ACDO}^{*}$.

As shown in Figure 5, with the increase in synergistic revenue, $S_{ACDO}^{*}$ decreases, and the probability that the initial probability of local government falls in the region ACDO also decreases, i.e., the probability that local governments choose (1,0) increases. Under System I, regardless of the dynamic carbon tax policy, local government A is more willing to choose the cooperative emission reduction strategy as cooperation benefits increase. That is, the government chooses to collaborate to reduce emissions in exchange for a higher benefit. Local government B, on the other hand, will refuse to cooperate. Although the benefit of cooperation increases the likelihood of the government opting for cooperative emission reduction to some extent, the net benefit of the government B opting for cooperative emission reduction may be far less than the benefit of opting for non-cooperative emission reduction. Therefore, the government will continue to refuse to cooperate.

### Analysis of Factors Affecting the Strategy of ‘Cooperation’

As previously stated, Chengdu has a lower forest coverage than Chongqing. Without sacrificing generality, the positive externalities brought about by cooperative emission reduction in Chongqing are greater [41], i.e., $\theta_{A}=9$ and $\theta_{B}=8$. Additionally, considering that the emission reduction technologies and other conditions of the two are the same, without losing generality, we assumed that $R_{A}=6$, $R_{B}=5$, $C_{A}^{}=4$, $C_{B}^{}=3$, $\pi_{s}=7$, $C_{s}=4$, and F=2. Therefore, we can further determine that the initial values of local governments in the two cases are $x_{0}=0.33$, $y_{0}=0.40$, $x_{1}=0.46$, and $y_{1}=0.62$.

**Figure 6:**
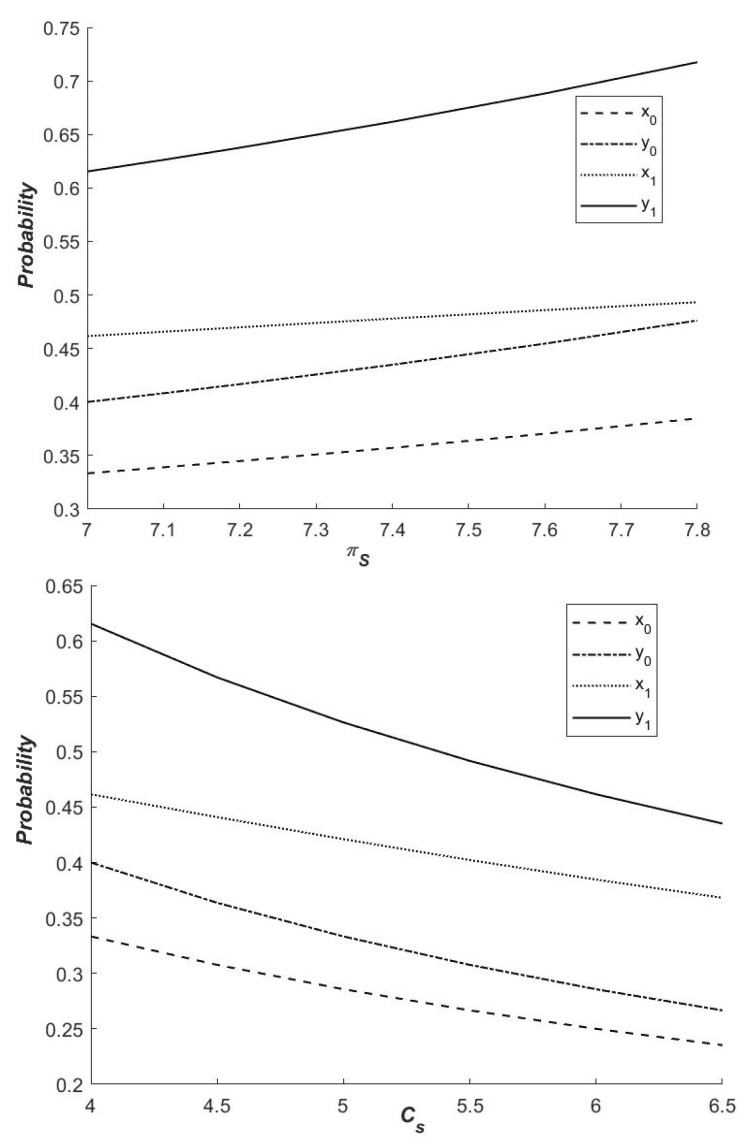
Influence of $\pi_{S}$ and $C_{s}$ on the probability of the strategy of ‘Cooperation’.

As illustrated in Figure 6, the probability of both governments choosing a cooperative emission reduction strategy is higher in the presence of a dynamic carbon tax than in the absence of a dynamic carbon tax. In other words, enacting a dynamic carbon tax policy can increase local governments’ willingness to collaborate on emission reductions. In both cases, the likelihood of choosing cooperative emission reduction strategies with the cooperation of local government revenue increases, and the increase of synergies can effectively promote cooperation between local governments, improving carbon emission efficiency. At the same time, the cost of intergovernmental cooperation is negatively related to the likelihood of intergovernmental cooperation in emission reduction. Therefore, the cost increase will reduce local governments’ willingness to pursue a “cooperative” strategy. It can be stated that increasing cooperation benefits or reducing cooperation costs and ensuring that the net benefits of local governments choosing cooperative emission reduction strategies are greater than those of non-cooperative emission reduction strategies can increase both governments’ willingness to cooperate in emission reduction and promote the development of regional low-carbon economies. Furthermore, the figure shows that, under the influence of different cooperation benefits and costs, local governments with varying resource endowments choose different growth and decline rates of cooperative emission reduction strategies.

**Figure 7:**
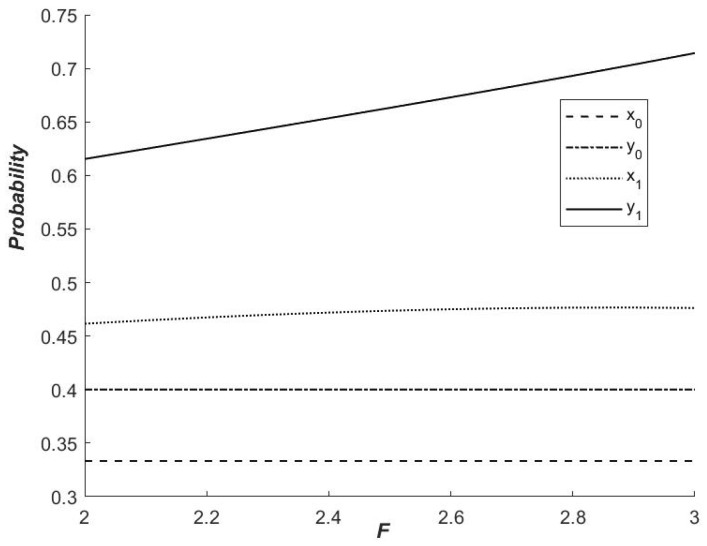
Influence of F on the probability of the strategy of ‘Cooperation’.

As illustrated in Figure 7, under the influence of a dynamic carbon tax, the likelihood of local governments opting for cooperative emission reduction is higher than under no dynamic carbon tax. That is, enacting the dynamic carbon tax policy can effectively increase both governments’ willingness to collaborate on emission reductions. At the same time, the carbon tax influences local government selection. As the carbon tax rises, so does the likelihood of a “cooperation” strategy. That is, as the carbon tax penalty increases, the likelihood of local governments cooperating in emission reduction increases. In short, the government’s dynamic carbon tax can effectively encourage local governments to implement collaborative emission-reduction strategies. Furthermore, dynamic carbon tax gives a larger role for low resources endowment government. The possible reason is that the government’s limited resource endowment means that the cost of cooperative emission reduction is higher, so it is more willing to pursue a non-cooperative emission reduction strategy. When the punishment is higher, the net benefits brought by local governments that choose not to cooperate are reduced, increasing the likelihood of choosing a cooperative emission reduction strategy.

**Figure 8:**
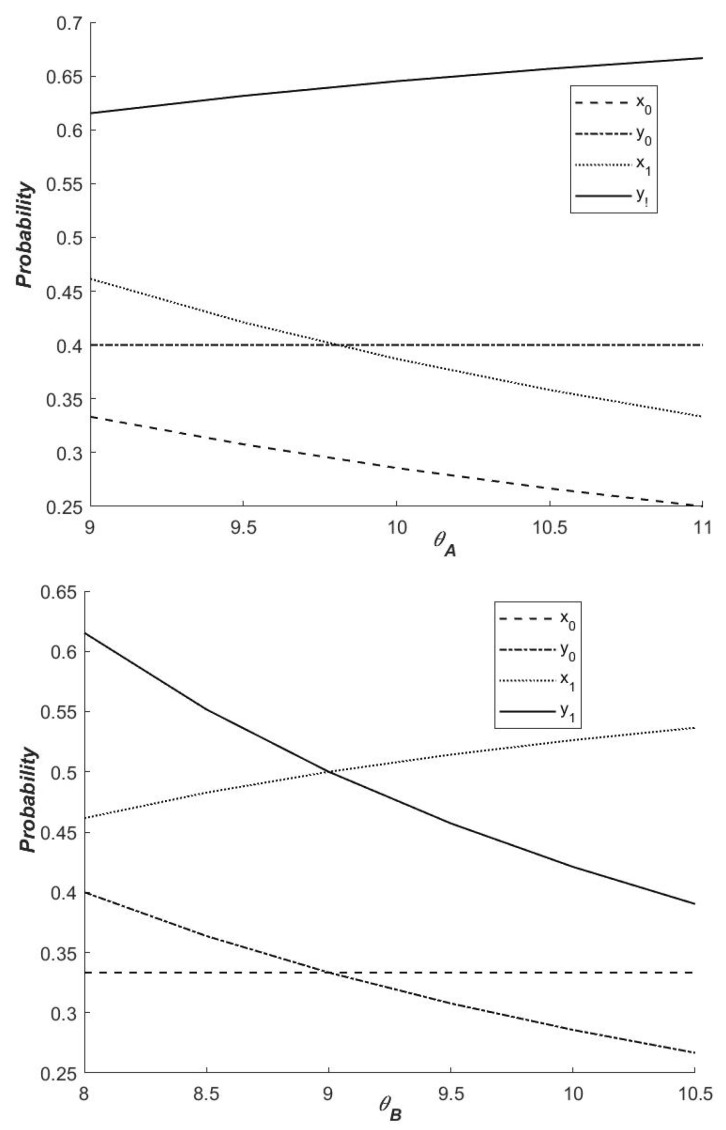
Influence of $\theta_{A}$ and $\theta_{B}$ on the probability of the strategy of ‘Cooperation’.

Figure 8 shows that whether or not there is a carbon tax policy, when the positive externality of a government’s cooperative emission reduction is greater, the government’s willingness to continue to choose cooperative emission reduction gradually decreases. That is, in both cases, when $\theta_{A}(\theta_{B})$ increases, the probability of government A(B) choosing a cooperative emission reduction will decrease. The reason for this is that the increase in positive externalities caused by the government’s choice of cooperative emission reduction strategy will reduce the government’s overall net benefits. The likelihood of local governments choosing a cooperative emission reduction strategy decreases as the net benefit decreases. When the net benefit is less than 0, the government abandons its original strategy and opts for non-cooperative emission reduction.

Without the influence of a dynamic carbon tax policy, the probability of local government B choosing cooperative emission reduction strategy almost remains unchanged when the positive external effect brought by cooperative emission reduction strategy increases. At this time, under the positive external effect brought by local government A’s cooperative emission reduction, the net benefit of local government B even if it chooses non-cooperative emission reduction is still much higher than that of cooperative emission reduction, so the probability of this government choosing cooperative emission reduction is nearly unchanged. However, if there is a dynamic carbon tax policy, the net benefit to local government B from higher punishment is less than the net benefit from cooperative emission reduction, and local government B will choose cooperative emission reduction. Simultaneously, when there is a dynamic carbon tax, the likelihood of local government *B* opting for a cooperative emission reduction strategy increases. It can be stated that the dynamic carbon tax policy directly influences the likelihood of local governments cooperating in emission reductions and increases both sides’ willingness to cooperate.

Similarly, when local government B selects the cooperative emission reduction strategy, the higher the positive external effect brought to local government A, the lower the willingness of government A to select the cooperative emission reduction strategy. One possible explanation is that the net benefits of local government A outweigh the benefits of cooperative emission reduction.

Clearly, implementing the dynamic carbon tax policy can effectively promote the two governments’ cooperation in emission reduction. To improve both governments’ willingness to cooperate, the central government should do everything possible to reduce the influence of externalities on both sides and provide carbon emission reduction subsidies to governments that choose cooperative emission reduction strategies.

## 8. Conclusions

In this study, we introduced the two scenarios of the presence or absence of carbon tax policy, built an evolutionary game model for multiple local governments to cooperate in emission reduction, and discussed the factors that affect governments’ willingness to cooperate in emission reduction. We found that, first, the benefits and costs of cooperation affect the willingness of local governments to cooperate in emission reduction. With the increase in synergistic benefits, the willingness of local governments to cooperate increases. However, under the influence of different cooperation benefits and costs, the probability of improving the government’s choice of cooperative emission reduction strategies is different. Second, in the process of cooperative carbon emission reduction, the willingness of governments to cooperate among themselves to reduce emissions is affected by externalities. The greater a government’s positive externality, the less willing the government is to choose cooperative emission reduction. On the contrary, people are more willing to work together to reduce emissions. Thus, in terms of government cooperation on carbon emission reduction, free-riding behavior reduces the effectiveness of bilateral emission reduction cooperation. However, if choosing the cooperative emission reduction strategy results in more benefits than negative externalities, the government will choose the cooperative emission reduction strategy. Third, the carbon tax policy can affect the probability that local governments will choose cooperative emission reduction and improve their willingness to cooperate in emission reduction. Additionally, different carbon taxes affect the willingness of the two sides to cooperate in emission reduction.

We found that due to the existence of information asymmetry and search costs, the benefits of local government cooperation in emission reduction decrease, and the enthusiasm for local government cooperation in emission reduction is also affected. Therefore, the central government should facilitate cooperation between local governments and directly invest or assist in establishing a regional emission reduction exchange platform, i.e., build an emission reduction information exchange platform and an emission reduction technology exchange platform, to help local governments achieve cooperative emission reduction. Second, the low willingness of local governments to reduce emissions is due to the external effects of carbon emissions and the emergence of government free-riding behavior. Therefore, the central government can comprehensively use administrative, legal, economic, and other means to internalize its external effects and enhance the willingness of local governments to cooperate in emission reduction. Third, the central government can further optimize the existing carbon tax system and maximize the willingness of local governments to cooperate among themselves to reduce emissions.

## Figures and Tables

**Table 1:** Notation.

Parameter	Description	Range
$C_{A}$	Costs of the government A choosing carbon emission reduction	$C_{A}>0$
$C_{B}$	Costs of the government B choosing carbon emission reduction	$C_{B}>0$
$C_{s}$	Costs of the government choosing ‘Cooperation’	$C_{s}>0$
$R_{A}$	Profits of the government A in case of the reduction of carbon emission	$R_{A}>0$
$R_{B}$	Profits of the government B in case of the reduction of carbon emission	$R_{B}>0$
$\pi_{s}$	Profits from the government choosing ‘Cooperation’	$\pi_{s}>0$
$\theta_{A}$	Profits that the government A brings to the government B in the process of the reduction of carbon emission	$\theta_{A}>0$
$\theta_{B}$	Profits that the government B brings to the government A in the process of the reduction of carbon emission	$\theta_{B}>0$
F	Carbon taxes from the government choosing ‘Not cooperation’	F>0
y	Probability that the government B chooses ‘Cooperation’	y∈[0,1]
x	Probability that the government A chooses ‘Cooperation’	x∈[0,1]

**Table 2:** Payoff matrix between the governments without carbon tax.

Subjects and Strategies		Government B	
		Cooperation y	Not Cooperation 1−y
Government A	Cooperation x	$R_{A}-C_{A}+\pi_{s}-C_{s}$ $,\;R_{B}-C_{B}+\pi_{s}-C_{s}$	$R_{A}-C_{A}-\theta_{B}$ $,\;\theta_{A}$
	Not cooperation 1−x	$\theta_{B}$ $,\;R_{B}-C_{B}-\theta_{A}$	$-\theta_{B}$ $,-\theta_{A}$

**Table 3:** Payoff matrix between the governments with dynamic carbon tax.

Subjects and Strategies		Government B	
		Cooperation y	Not Cooperation 1−y
Government A	Cooperation x	$R_{A}-C_{A}+\pi_{s}-C_{s}$, $R_{B}-C_{B}+\pi_{s}-C_{s}$	$R_{A}-C_{A}-\theta_{B}$, $\theta_{A}-F(1-y)$
	Not Cooperation 1−x	$\theta_{B}-F(1-x)$, $R_{B}-C_{B}-\theta_{A}$	$-\theta_{B}-F(1-x)$, $-\theta_{A}-F(1-y)$

**Table 4:** Stability analysis of equilibrium points in the situation of a dynamic carbon tax.

Condition 1	Points	*Det*	*Tr*	Stability	Condition 2	Points	*Det*	*Tr*	Stability	Condition 3	Points	*Det*	*Tr*	Stability
*g_1_ < −F < 0* *g_2_ < −F < 0*	(0,0)	+	+	Unstable point	*g_1_ < −F < 0* *−F < g_2_ < 0*	(0,0)	+	+	Unstable point	*g_1_ < −F < 0* *g_2_ > 0*	(0,0)	+	+	Unstable point
	(0,1)	+	−	ESS		(0,1)	+	−	ESS		(0,1)	+	−	ESS
	(1,1)	+	+	Unstable point		(1,1)	+	+	Unstable point		(1,1)	−	?	Saddle point
	(1,0)	+	−	ESS		(1,0)	−	?	Saddle point		(1,0)	−	?	Saddle point
Condition 4	Points	*Det*	*Tr*	Stability	Condit-ion 5	Points	*Det*	*Tr*	Stability	Condit-ion 6	Points	*Det*	*Tr*	Stability
*−F < g_1_ < 0* *g_2_ < −F < 0*	(0,0)	+	+	Unstable point	*−F < g_1_ < 0* *−F < g_2_ < 0*	(0,0)	+	+	Unstable point	*−F < g_1_ < 0* *g_2_ > 0*	(0,0)	+	+	Unstable point
	(0,1)	−	?	Saddle point		(0,1)	−	?	Saddle point		(0,1)	−	?	Saddle point
	(1,1)	+	+	Unstable point		(1,1)	+	+	Unstable point		(1,1)	−	?	Saddle point
	(1,0)	+	−	ESS		(1,0)	−	?	Saddle point		(1,0)	−	?	Saddle point
Condition 7	Points	*Det*	*Tr*	Stability	Condit-ion 8	Points	*Det*	*Tr*	Stability	Condit-ion 9	Points	*Det*	*Tr*	Stability
*g_1_ > 0* *g_2_ < −F < 0*	(0,0)	+	+	Unstable point	*g_1_ > 0* *−F < g_2_ < 0*	(0,0)	+	+	Unstable point	*g_1_ > 0* *g_2_ > 0*	(0,0)	+	+	Unstable point
	(0,1)	−	?	Saddle point		(0,1)	−	?	Saddle point		(0,1)	−	?	Saddle point
	(1,1)	−	?	Saddle point		(1,1)	−	?	Saddle point		(1,1)	+	−	ESS
	(1,0)	+	-	ESS		(1,0)	−	?	Saddle point		(1,0)	−	?	Saddle point

**Table 5:** A payoff matrix between the governments without a carbon tax.

Subjects and Strategies		Government B	Government C	
			Cooperation z	Not Cooperation 1−z
Government A	Cooperation x	Cooperation y	$R_{A}-C_{A}+\pi_{s}-C_{s}$, $R_{B}-C_{B}+\gamma \left(\pi_{s}-C_{s}\right)$, $R_{C}-C_{C}+\pi_{s}-C_{s}$	$R_{A}-C_{A}+\pi_{s}-C_{s}$, $R_{B}-C_{B}+\pi_{s}-C_{s}-\theta_{C}$, $\theta_{B}$
		Not cooperation 1−y	$R_{A}-C_{A}-\theta_{B}$, $\theta_{C}+\theta_{A}$, $R_{C}-C_{C}-\theta_{B}$	$R_{A}-C_{A}-\theta_{B}$, $\theta_{A}-\theta_{C}$, $-\theta_{B}$
	Cooperation 1−x	Cooperation y	$\theta_{B}$, $R_{B}-C_{B}+\pi_{s}-C_{s}-\theta_{A}$, $R_{C}-C_{C}+\pi_{s}-C_{s}$	$\theta_{B}$, $R_{B}-C_{B}-\theta_{C}-\theta_{A}$, $\theta_{B}$
		Not cooperation 1−y	$-\theta_{B}$, $\theta_{C}-\theta_{A}$, $R_{C}-C_{C}-\theta_{B}$	$-\theta_{B}$, $-\theta_{C}-\theta_{A}$, $-\theta_{B}$

**Table 6:** Eigenvalues and the evolutionary stability of equilibrium points.

	Equilibrium Point	Eigenvalue	Evolutionary Stability
Case 1	(0,0,0)	$\begin{array}{c} \lambda_{1}=R_{A}-C_{A}^{}\\ \lambda_{2}=R_{B}-C_{B}^{}\\ \lambda_{3}=R_{C}-C_{C}^{} \end{array}$	Unstable point
Case 2	(0,0,1)	$\begin{array}{c} \lambda_{1}=R_{A}-C_{A}^{}\\ \lambda_{2}=R_{B}-C_{B}^{}+\pi_{s}-C_{s}-\theta_{C}\\ \lambda_{3}=C_{C}^{}-R_{C} \end{array}$	Saddle point
Case 3	(0,1,0)	$\begin{array}{c} \lambda_{1}=R_{A}-C_{A}^{}+\pi_{s}-C_{s}-\theta_{B}\\ \lambda_{2}=C_{B}^{}-R_{B}\\ \lambda_{3}=R_{C}-C_{C}^{}+\pi_{s}-C_{s}-\theta_{B} \end{array}$	Saddle point
Case 4	(1,0,0)	$\begin{array}{c} \lambda_{1}=C_{A}^{}-R_{A}\\ \lambda_{2}=R_{B}-C_{B}^{}-\theta_{A}+\pi_{s}-C_{s}\\ \lambda_{3}=R_{C}-C_{C}^{} \end{array}$	Saddle point
Case 5	(0,1,1)	$\lambda_{1}=R_{A}-C_{A}^{}+\pi_{s}-C_{s}-\theta_{B}$ $\lambda_{2}=-\left[R_{B}-C_{B}^{}-\theta_{A}+\pi_{s}-C_{s}\right]$ $\lambda_{3}=-\left[R_{C}-C_{C}^{}+\pi_{s}-C_{s}-\theta_{B}\right]$	$\mathrm{If}\;\lambda_{1}=R_{A}-C_{A}^{}+\pi_{s}-C_{s}-\theta_{B}<0$ $\lambda_{2}=-\left[R_{B}-C_{B}^{}-\theta_{A}+\pi_{s}-C_{s}\right]<0$ $\lambda_{3}=-\left[R_{C}-C_{C}^{}+\pi_{s}-C_{s}-\theta_{B}\right]<0$ exist simultaneously, an equilibrium point exists.
Case 6	(1,1,0)	$\lambda_{1}=-\left[R_{A}-C_{A}^{}+\pi_{s}-C_{s}-\theta_{B}\right]$ $\lambda_{2}=-\left[R_{B}-C_{B}^{}-\theta_{A}+\pi_{s}-C_{s}\right]$ $\lambda_{3}=R_{C}-C_{C}^{}+\pi_{s}-C_{s}-\theta_{B}$	If $\lambda_{1}=-\left[R_{A}-C_{A}^{}+\pi_{s}-C_{s}-\theta_{B}\right]<0$ $\lambda_{2}=-\left[R_{B}-C_{B}^{}-\theta_{A}+\pi_{s}-C_{s}\right]<0$ $\lambda_{3}=R_{C}-C_{C}^{}+\pi_{s}-C_{s}-\theta_{B}<0$ exist simultaneously, an equilibrium point exists.
Case 7	(1,0,1)	$\lambda_{1}=C_{A}^{}-R_{A}$ $\lambda_{2}=R_{B}-C_{B}^{}-\theta_{A}-\theta_{C}+\gamma \left(\pi_{s}-C_{s}\right)$ $\lambda_{3}=C_{C}^{}-R_{C}$	$\mathrm{If}\;\lambda_{2}=R_{B}-C_{B}^{}-\theta_{A}-\theta_{C}+\gamma \left(\pi_{s}-C_{s}\right)<0$ are satisfied, a stable point exists.
Case 8	(1,1,1)	$\lambda_{3}=-\left[R_{C}-C_{C}^{}+\pi_{s}-C_{s}-\theta_{B}\right]$ $\lambda_{2}=-\left[R_{B}-C_{B}^{}-\theta_{A}-\theta_{C}+\gamma \left(\pi_{s}-C_{s}\right)\right]$ $\lambda_{3}=-\left[R_{C}-C_{C}^{}+\pi_{s}-C_{s}-\theta_{B}\right]$	If $\lambda_{1}=-\left[R_{A}-C_{A}^{}+\pi_{s}-C_{s}-\theta_{B}\right]<0$ $\lambda_{2}=-\left[R_{B}-C_{B}^{}-\theta_{A}-\theta_{C}+\gamma \left(\pi_{s}-C_{s}\right)\right]<0$ $\lambda_{3}=-\left[R_{C}-C_{C}^{}+\pi_{s}-C_{s}-\theta_{B}\right]<0$ are satisfied simultaneously, a stable point exists.
